# (*Z*)-3-Chloro­methyl­idene-5,6-dimeth­oxy-2-methyl-2,3-dihydro-1,2-benzothia­zole 1,1-dioxide

**DOI:** 10.1107/S1600536810049561

**Published:** 2010-12-04

**Authors:** Jatinder P. Bassin, Virender P. Shah, Lee Martin, William Clegg, Ross W. Harrington

**Affiliations:** a School of Pharmacy, University of Hertfordshire, College Lane, Hatfield AL10 9AB, England; b School of Chemistry, Newcastle University, Newcastle upon Tyne NE1 7RU, England

## Abstract

The title compound, C_11_H_12_ClNO_4_S, adopts a *Z* configuration about the C=C double bond. The benzisothia­zole system is essentially planar [maximum deviation of 0.235 (2) Å for the S atom]. In the crystal, the mol­ecules stack parallel to each other in the *b*-axis direction, with inter­planar spacings for the benzene and thia­zole rings ranging from 3.402 (2) to 3.702 (2) Å.

## Related literature

3-Substituted 1,2-benzisothia­zole-1,1-dioxides are an important class of heterocycles with a broad range of biological activity, see: Feit *et al.* (1973 ▶); Shutske *et al.* (1983 ▶); Bachman *et al.* (1978 ▶); Vicini *et al.* (2003 ▶); Sharmeen *et al.* (2001 ▶). Various synthetic routes have been developed for the synthesis of 1,2-benzisothia­zole-1,1-dioxides, see: Chapman & Peart (1996 ▶). Carbonation of *ortho*-lithia­ted sulfonamides is the most common method for the preparation of substituted saccharins; however, this results in poor yields (Lombardino, 1971 ▶) and is limited by the availability of starting materials. A recent improved synthesis of 1,2-benzisothia­zole-1,1-dioxides involved cyclization of *N*-acyl-benzene­sulfonamides using LDA, see: Hermann *et al.* (1992 ▶).
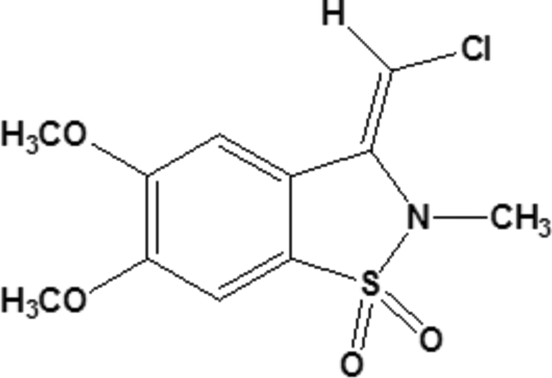


## Experimental

### 

#### Crystal data

C_11_H_12_ClNO_4_S
*M* *_r_* = 289.73
Triclinic, 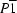

*a* = 7.578 (5) Å
*b* = 7.904 (6) Å
*c* = 10.002 (7) Å
α = 88.875 (11)°
β = 86.293 (10)°
γ = 84.176 (11)°
*V* = 594.7 (7) Å^3^
*Z* = 2
Synchrotron radiation
λ = 0.6937 Å
μ = 0.50 mm^−1^
*T* = 120 K
0.20 × 0.04 × 0.01 mm

#### Data collection

Bruker APEXII CCD diffractometer
Absorption correction: multi-scan (*SADABS*; Sheldrick, 2002 ▶) *T*_min_ = 0.905, *T*_max_ = 0.990
4651 measured reflections
2237 independent reflections
1671 reflections with *I* > 2σ(*I*)
*R*_int_ = 0.038

#### Refinement

*R*[*F* ^2^ > 2σ(*F* ^2^)] = 0.065
*wR*(*F* ^2^) = 0.177
*S* = 1.02
2237 reflections
167 parameters
H-atom parameters constrained
Δρ_max_ = 1.29 e Å^−3^
Δρ_min_ = −0.43 e Å^−3^

### 

Data collection: *APEX2* (Bruker, 2004 ▶); cell refinement: *SAINT* (Bruker, 2004 ▶); data reduction: *SAINT*; program(s) used to solve structure: *SHELXTL* (Sheldrick, 2008 ▶); program(s) used to refine structure: *SHELXTL*; molecular graphics: *SHELXTL*; software used to prepare material for publication: *SHELXTL* and local programs.

## Supplementary Material

Crystal structure: contains datablocks I, global. DOI: 10.1107/S1600536810049561/jh2234sup1.cif

Structure factors: contains datablocks I. DOI: 10.1107/S1600536810049561/jh2234Isup2.hkl

Additional supplementary materials:  crystallographic information; 3D view; checkCIF report

## Figures and Tables

**Fig. 1.**
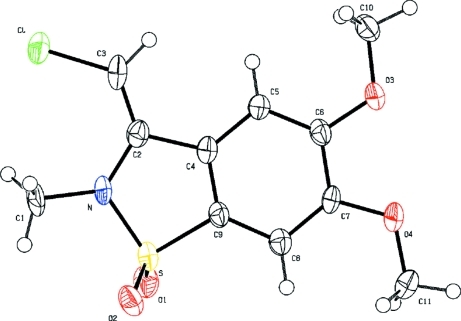
Ellipsoid plot.

## supplementary crystallographic information

### Experimental

The title compound was synthesized by reacting 2-(5,6-dimethoxy-2-methyl-1,1-dioxido-2,3-dihydrobenzo [*d*]isothiazol-3-yl)-1-phenylethanone (1 g; 2.77 mmol), dissolved in pyridine (10 ml), with sodium hypochlorite (10 ml). An exothermic reaction occurred and with time the solution became turbid. The reaction mixture was stirred for a total of 30 minutes and then poured onto crushed ice. The resulting light yellow solid was filtered by suction filtration and air dried. It was re-crystallized from ethanol to afford a creamy white solid which was re-crystallized a second time to give 1 as a colourless crystalline product [yield: 87%, m.p.: 457 K].

### Refinement

H atoms were constrained with a riding model, having C—H = 0.95–0.98 Å and *U*_iso_(H) = 1.5_eq_(H) for methyl groups and 1.2*U*_eq_(C) for other atoms. The largest residual electron density peak lies 1.81 Å from Cl1 and 1.04 Å from C3, but has no structural chemical significance.

### Crystal data

**Table:**

C_11_H_12_ClNO_4_S	*Z* = 2
*M**_r_* = 289.73	*F*(000) = 300
Triclinic, *P*1	*D*_x_ = 1.618 Mg m^−^^3^
Hall symbol: -P 1	Synchrotron radiation, λ = 0.6937 Å
*a* = 7.578 (5) Å	Cell parameters from 974 reflections
*b* = 7.904 (6) Å	θ = 2.6–24.5°
*c* = 10.002 (7) Å	µ = 0.50 mm^−^^1^
α = 88.875 (11)°	*T* = 120 K
β = 86.293 (10)°	Needle, colourless
γ = 84.176 (11)°	0.20 × 0.04 × 0.01 mm
*V* = 594.7 (7) Å^3^	

### Data collection

**Table:**

Bruker APEXII CCD diffractometer	2237 independent reflections
Radiation source: Daresbury SRS station 9.8	1671 reflections with *I* > 2σ(*I*)
silicon 111	*R*_int_ = 0.038
thin–slice ω scans	θ_max_ = 25.0°, θ_min_ = 2.0°
Absorption correction: multi-scan (*SADABS*; Sheldrick, 2002)	*h* = −9→9
*T*_min_ = 0.905, *T*_max_ = 0.990	*k* = −9→9
4651 measured reflections	*l* = −12→12

### Refinement

**Table:**

Refinement on *F*^2^	Secondary atom site location: difference Fourier map
Least-squares matrix: full	Hydrogen site location: inferred from neighbouring sites
*R*[*F*^2^ > 2σ(*F*^2^)] = 0.065	H-atom parameters constrained
*wR*(*F*^2^) = 0.177	*w* = 1/[σ^2^(*F*_o_^2^) + (0.0319*P*)^2^ + 1.6618*P*] where *P* = (*F*_o_^2^ + 2*F*_c_^2^)/3
*S* = 1.02	(Δ/σ)_max_ < 0.001
2237 reflections	Δρ_max_ = 1.29 e Å^−^^3^
167 parameters	Δρ_min_ = −0.43 e Å^−^^3^
0 restraints	Extinction correction: *SHELXTL* (Sheldrick, 2008), Fc^*^=kFc[1+0.001xFc^2^λ^3^/sin(2θ)]^-1/4^
Primary atom site location: structure-invariant direct methods	Extinction coefficient: 0.084 (15)

### Special details

**Table:**

Geometry. All e.s.d.'s (except the e.s.d. in the dihedral angle between two l.s. planes) are estimated using the full covariance matrix. The cell e.s.d.'s are taken into account individually in the estimation of e.s.d.'s in distances, angles and torsion angles; correlations between e.s.d.'s in cell parameters are only used when they are defined by crystal symmetry. An approximate (isotropic) treatment of cell e.s.d.'s is used for estimating e.s.d.'s involving l.s. planes.
Refinement. Refinement of *F*^2^ against ALL reflections. The weighted *R*-factor *wR* and goodness of fit *S* are based on *F*^2^, conventional *R*-factors *R* are based on *F*, with *F* set to zero for negative *F*^2^. The threshold expression of *F*^2^ > σ(*F*^2^) is used only for calculating *R*-factors(gt) *etc*. and is not relevant to the choice of reflections for refinement. *R*-factors based on *F*^2^ are statistically about twice as large as those based on *F*, and *R*- factors based on ALL data will be even larger.

### Fractional atomic coordinates and isotropic or equivalent isotropic displacement parameters (Å^2^)

**Table:**

	*x*	*y*	*z*	*U*_iso_*/*U*_eq_	
S1	0.20714 (16)	0.29186 (15)	0.38990 (10)	0.0286 (4)	
Cl1	0.72928 (18)	0.01424 (17)	0.14119 (11)	0.0402 (4)	
N1	0.3721 (5)	0.2029 (5)	0.2891 (3)	0.0315 (9)	
C1	0.3515 (8)	0.2190 (8)	0.1449 (4)	0.0441 (14)	
H1A	0.4303	0.3002	0.1058	0.066*	
H1B	0.2279	0.2595	0.1290	0.066*	
H1C	0.3823	0.1079	0.1031	0.066*	
C2	0.5330 (6)	0.1725 (6)	0.3483 (4)	0.0277 (10)	
C3	0.6901 (7)	0.0988 (6)	0.2982 (4)	0.0353 (12)	
H3	0.7873	0.0931	0.3541	0.042*	
C4	0.5098 (6)	0.2358 (5)	0.4871 (4)	0.0255 (10)	
C5	0.6343 (6)	0.2194 (6)	0.5848 (4)	0.0270 (10)	
H5	0.7509	0.1660	0.5653	0.032*	
C6	0.5834 (6)	0.2829 (6)	0.7102 (4)	0.0268 (10)	
C7	0.4134 (6)	0.3697 (6)	0.7387 (4)	0.0255 (10)	
C8	0.2894 (6)	0.3844 (6)	0.6435 (4)	0.0271 (10)	
H8	0.1733	0.4400	0.6615	0.033*	
C9	0.3435 (6)	0.3132 (6)	0.5189 (4)	0.0250 (10)	
C10	0.8610 (6)	0.1808 (6)	0.7939 (5)	0.0328 (11)	
H10A	0.8510	0.0663	0.7612	0.049*	
H10B	0.9205	0.1726	0.8782	0.049*	
H10C	0.9306	0.2431	0.7271	0.049*	
C11	0.2152 (7)	0.5175 (7)	0.8980 (5)	0.0354 (11)	
H11A	0.1882	0.6091	0.8329	0.053*	
H11B	0.2138	0.5654	0.9878	0.053*	
H11C	0.1258	0.4362	0.8971	0.053*	
O1	0.0829 (5)	0.1725 (4)	0.4255 (3)	0.0371 (8)	
O2	0.1341 (5)	0.4480 (4)	0.3321 (3)	0.0358 (8)	
O3	0.6882 (4)	0.2685 (4)	0.8156 (3)	0.0316 (8)	
O4	0.3860 (4)	0.4328 (4)	0.8638 (3)	0.0320 (8)	

### Atomic displacement parameters (Å^2^)

**Table:**

	*U*^11^	*U*^22^	*U*^33^	*U*^12^	*U*^13^	*U*^23^
S1	0.0357 (7)	0.0326 (7)	0.0185 (6)	−0.0065 (5)	−0.0058 (5)	0.0001 (4)
Cl1	0.0493 (8)	0.0498 (8)	0.0204 (6)	0.0003 (6)	−0.0013 (5)	−0.0052 (5)
N1	0.039 (2)	0.043 (2)	0.0123 (17)	−0.0009 (18)	−0.0043 (16)	−0.0005 (16)
C1	0.052 (3)	0.068 (4)	0.012 (2)	−0.006 (3)	−0.008 (2)	0.002 (2)
C2	0.036 (3)	0.027 (2)	0.020 (2)	−0.003 (2)	0.0002 (18)	0.0020 (18)
C3	0.054 (3)	0.036 (3)	0.017 (2)	−0.010 (2)	−0.003 (2)	−0.0034 (19)
C4	0.033 (2)	0.026 (2)	0.018 (2)	−0.0053 (19)	−0.0008 (18)	−0.0002 (17)
C5	0.028 (2)	0.032 (2)	0.020 (2)	−0.0014 (19)	−0.0004 (18)	0.0004 (18)
C6	0.029 (2)	0.034 (2)	0.019 (2)	−0.0067 (19)	−0.0039 (17)	0.0038 (18)
C7	0.034 (2)	0.029 (2)	0.0132 (19)	−0.0050 (19)	0.0020 (17)	−0.0001 (16)
C8	0.030 (2)	0.030 (2)	0.022 (2)	−0.0059 (19)	−0.0021 (18)	0.0008 (18)
C9	0.029 (2)	0.029 (2)	0.017 (2)	−0.0033 (19)	−0.0032 (17)	0.0023 (17)
C10	0.034 (3)	0.036 (3)	0.029 (2)	−0.001 (2)	−0.011 (2)	0.001 (2)
C11	0.039 (3)	0.042 (3)	0.024 (2)	−0.002 (2)	0.005 (2)	−0.006 (2)
O1	0.041 (2)	0.042 (2)	0.0306 (18)	−0.0136 (16)	−0.0048 (15)	−0.0041 (15)
O2	0.044 (2)	0.0359 (19)	0.0287 (17)	−0.0017 (15)	−0.0140 (15)	0.0039 (14)
O3	0.0333 (18)	0.0432 (19)	0.0182 (15)	−0.0007 (15)	−0.0065 (13)	−0.0009 (13)
O4	0.0385 (19)	0.0414 (19)	0.0159 (15)	−0.0035 (15)	−0.0008 (13)	−0.0020 (13)

### Geometric parameters (Å, °)

**Table:**

S1—N1	1.661 (4)	C5—C6	1.377 (6)
S1—C9	1.725 (4)	C6—C7	1.411 (7)
S1—O1	1.424 (4)	C6—O3	1.357 (5)
S1—O2	1.427 (3)	C7—C8	1.376 (6)
Cl1—C3	1.715 (5)	C7—O4	1.352 (5)
N1—C1	1.463 (5)	C8—H8	0.950
N1—C2	1.387 (6)	C8—C9	1.398 (6)
C1—H1A	0.980	C10—H10A	0.980
C1—H1B	0.980	C10—H10B	0.980
C1—H1C	0.980	C10—H10C	0.980
C2—C3	1.342 (7)	C10—O3	1.424 (6)
C2—C4	1.478 (6)	C11—H11A	0.980
C3—H3	0.950	C11—H11B	0.980
C4—C5	1.397 (6)	C11—H11C	0.980
C4—C9	1.365 (6)	C11—O4	1.420 (6)
C5—H5	0.950		
			
N1—S1—C9	93.3 (2)	C5—C6—C7	121.5 (4)
N1—S1—O1	110.3 (2)	C5—C6—O3	124.1 (4)
N1—S1—O2	109.9 (2)	C7—C6—O3	114.4 (4)
C9—S1—O1	110.6 (2)	C6—C7—C8	120.6 (4)
C9—S1—O2	115.1 (2)	C6—C7—O4	114.7 (4)
O1—S1—O2	115.4 (2)	C8—C7—O4	124.7 (4)
S1—N1—C1	117.1 (3)	C7—C8—H8	121.8
S1—N1—C2	114.2 (3)	C7—C8—C9	116.3 (4)
C1—N1—C2	125.1 (4)	H8—C8—C9	121.8
N1—C1—H1A	109.5	S1—C9—C4	110.2 (3)
N1—C1—H1B	109.5	S1—C9—C8	125.5 (4)
N1—C1—H1C	109.5	C4—C9—C8	124.1 (4)
H1A—C1—H1B	109.5	H10A—C10—H10B	109.5
H1A—C1—H1C	109.5	H10A—C10—H10C	109.5
H1B—C1—H1C	109.5	H10A—C10—O3	109.5
N1—C2—C3	129.9 (4)	H10B—C10—H10C	109.5
N1—C2—C4	108.7 (4)	H10B—C10—O3	109.5
C3—C2—C4	121.4 (4)	H10C—C10—O3	109.5
Cl1—C3—C2	125.2 (4)	H11A—C11—H11B	109.5
Cl1—C3—H3	117.4	H11A—C11—H11C	109.5
C2—C3—H3	117.4	H11A—C11—O4	109.5
C2—C4—C5	127.5 (4)	H11B—C11—H11C	109.5
C2—C4—C9	113.3 (4)	H11B—C11—O4	109.5
C5—C4—C9	119.3 (4)	H11C—C11—O4	109.5
C4—C5—H5	120.9	C6—O3—C10	117.2 (4)
C4—C5—C6	118.2 (4)	C7—O4—C11	116.7 (4)
H5—C5—C6	120.9		
			
C9—S1—N1—C1	−155.2 (4)	C5—C6—C7—O4	−176.5 (4)
C9—S1—N1—C2	4.6 (4)	O3—C6—C7—C8	−176.0 (4)
O1—S1—N1—C1	91.4 (4)	O3—C6—C7—O4	3.6 (6)
O1—S1—N1—C2	−108.8 (3)	C6—C7—C8—C9	−1.5 (6)
O2—S1—N1—C1	−37.0 (4)	O4—C7—C8—C9	178.9 (4)
O2—S1—N1—C2	122.8 (3)	C2—C4—C9—S1	6.2 (5)
S1—N1—C2—C3	177.8 (4)	C2—C4—C9—C8	−179.5 (4)
S1—N1—C2—C4	−1.8 (5)	C5—C4—C9—S1	−171.9 (3)
C1—N1—C2—C3	−24.3 (8)	C5—C4—C9—C8	2.5 (7)
C1—N1—C2—C4	156.1 (5)	C7—C8—C9—S1	171.8 (3)
N1—C2—C3—Cl1	−2.1 (7)	C7—C8—C9—C4	−1.6 (7)
C4—C2—C3—Cl1	177.5 (3)	N1—S1—C9—C4	−6.2 (3)
N1—C2—C4—C5	174.9 (4)	N1—S1—C9—C8	179.6 (4)
N1—C2—C4—C9	−2.9 (5)	O1—S1—C9—C4	107.0 (3)
C3—C2—C4—C5	−4.8 (7)	O1—S1—C9—C8	−67.3 (4)
C3—C2—C4—C9	177.4 (4)	O2—S1—C9—C4	−120.0 (3)
C2—C4—C5—C6	−177.8 (4)	O2—S1—C9—C8	65.8 (4)
C9—C4—C5—C6	0.0 (6)	C5—C6—O3—C10	−0.5 (6)
C4—C5—C6—C7	−3.1 (7)	C7—C6—O3—C10	179.4 (4)
C4—C5—C6—O3	176.8 (4)	C6—C7—O4—C11	−178.5 (4)
C5—C6—C7—C8	3.9 (7)	C8—C7—O4—C11	1.1 (6)
